# Lymph Node Metastasis, a Unique Independent Prognostic Factor in Early Gastric Cancer

**DOI:** 10.1371/journal.pone.0129531

**Published:** 2015-07-08

**Authors:** Bai-Wei Zhao, Yong-Ming Chen, Shan-Shan Jiang, Yin-Bo chen, Zhi-Wei Zhou, Yuan-Fang Li

**Affiliations:** 1 Sun Yat-sen University Cancer Center, State Key Laboratory of Oncology in South China, Collaborative Innovation Center for Cancer Medicine, Guangzhou, 510060, China; 2 Department of Gastric & pancreatic Surgery, Cancer Center, Sun Yat-Sen University, Guangzhou, China; INRS, CANADA

## Abstract

**Background:**

Lymph node metastasis (LNM) has been shown to be related to the prognosis of early gastric cancer (EGC). The choice of optimal treatment depends on an accurate pre-operative assessment of LNM status in EGC patients. However, in China, where EGC cases account for only a small part of gastric cancer (GC) cases, there are not enough data to make an accurate assessment. Therefore, this study, which involved a relatively large number of EGC patients, aimed to explore the relationship between clinicopathological characteristics and LNM in EGC.

**Methods:**

Clinicopathological data from 205 EGC patients who underwent surgical resection at Sun Yat-Sen University Cancer Center from January 2000 to December 2011 were retrospectively analyzed. Clinicopathological characteristics were assessed to identify effective predictive factors for LNM and overall survival.

**Results:**

LNM occurred in 52 (25.37%) EGC cases; of these cases, 18 occurred in intra-mucosal cancers (13 N1, 4 N2 and 1 N3), and 34 occurred in sub-mucosal cancers (22 N1, 7 N2 and 5 N3). Logistic regression analysis demonstrated that tumor differentiation (P=0.002), depth of tumor infiltration (P=0.004), vessel invasion (P=0.012), tumor size (P=0.020) and gender (P=0.022) were risk factors associated with LNM in EGC, listed in order of priority. The overall survival rate was 90.2%. Kaplan-Meier survival analysis showed that overall survival of EGC patients was significantly correlated with LNM (P=0.001), N staging (P<0.001) and invasion of lymphatic or blood vessels (P=0.010), but it was not correlated with tumor size, depth of tumor infiltration or tumor cell differentiation. Moreover, a multiple Cox regression analysis demonstrated that only N staging (P=0.001) could serve as an independent prognostic predictor in EGC patients.

**Conclusions:**

Because LNM independently predicts the prognosis of EGC, endoscopic mucosal resection (EMR) or endoscopic submucosal dissection (ESD) and laparoscopic partial gastrectomy should be cautiously used in high-risk EGC patients. A pre-operative assessment of LNM status based on clinicopathological factors may be useful for therapy planning.

## Introduction

**Fig 1:**
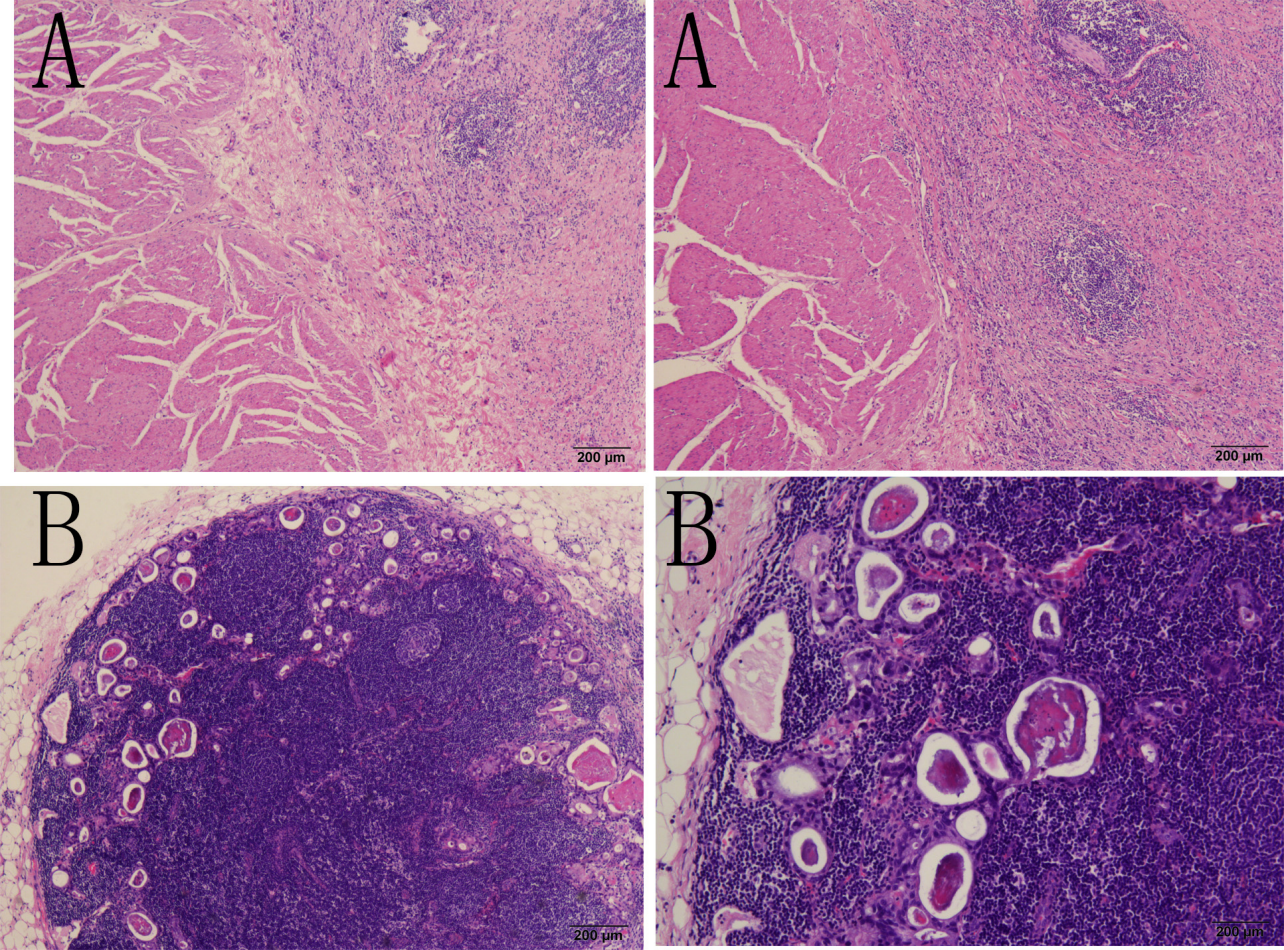
LNM occurred in an intra-mucosal gastric cancer patient shown in the H-E staining graph.

**Fig 2:**
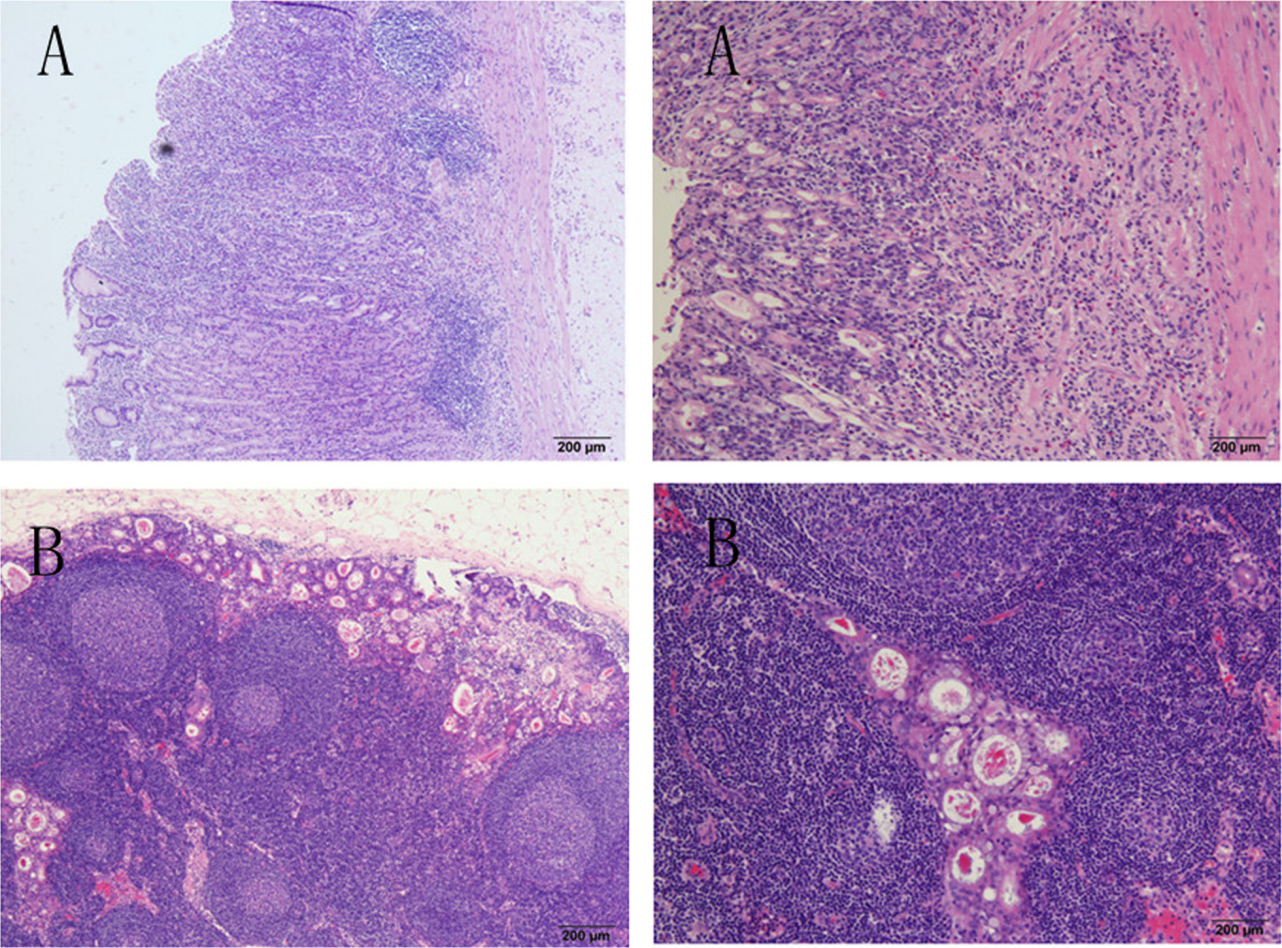
LNM occurred in a sub-mucosal gastric cancer patient shown in the H-E staining graph.

Early gastric cancer (EGC) is a gastric cancer in which the lesion is confined to the mucosa and submucosa, regardless of the tumor size or the status of lymph node metastasis (LNM) [1]. Compared with advanced gastric cancer (AGC), EGC patients have a better post-operation prognosis, with an overall survival rate as high as 90%[1]. EGC treatment consists of endoscopic mucosal resection (EMR) or endoscopic sub-mucosal dissection (ESD) and gastrectomy plus D1 or D2 lymphadenectomy through laparoscopic or open operation [2–4]. As reported in previous studies, LNM rarely occurs in intramucosal gastric cancers (usually in less than 6% of such cases). However, when the tumor invades into the sub-mucosa layer of the stomach wall in which lymphatic vessels are abundant, the rate of LNM increases significantly to above 10% [5] and the prognosis is relatively poor (Figs 1 and 2). Moreover, in some large-scale studies performed in Japan and Korea, the overall survival rate of lymph node-positive EGC fell to 70%-80%, and the relapse rate rose to 8%[6–8]. The use of radical surgery depends on the status of LNM. Thus, it is essential to summarize the clinicopathological characteristics of EGC patients to find the risk factors for LNM and to indicate an effective treatment for EGC patients.

China has a relatively high incidence of gastric cancer, but there are not sufficient data about ECG, due to delayed diagnoses. The present study involved a relatively large number of EGC patients and retrospectively investigated the relationship between the clinicopathologic factors of these EGC patients and LNM. We are looking forward to demonstrating the risk factors for LNM in EGC patients and choosing the optimal operation method (with or without lymphadenectomy) based on these factors.

## Methods

### Ethics statement

This research was approved by the Ethics Committee of the Sun Yat-sen University Cancer Center, and written informed consent was obtained from each patient involved in the study.

### Patients

From January 2000 to December 2011, clinicopathological data from 2,264 gastric cancer patients who were treated at Sun Yat-sen University Cancer Center were retrospectively analyzed. All 2,264 cases were diagnosed by routine pathological examination,. Two hundred eight (9.18%) of these cases were diagnosed as T1 gastric cancer (GC). Of the T1 patients, 3 cases (1.44% of the total) were excluded as a result of distant metastasis (2 liver metastasis and 1 peritoneal nodule). In all, 205 EGC patients who met the following eligibility criteria were involved in the present study: (1) diagnosis of early gastric cancer (i.e., T1a or T1b, N0-3, M0) identified by histopathological examination according to the 7th edition of the International Union Against Cancer (UICC) Tumor-Node-Metastasis (TNM) staging system; (2) surgical history that included gastrectomy plus lymphadenectomy (D1 or D2); (3) availability of complete follow-up data (follow-up visits ended on December 31st, 2012, and the survival period was calculated from the date of surgery to the end of the follow-up or the date of death due to relapse or metastasis); (4) no preoperative treatment, such as chemotherapy or radiotherapy; (5) no history of familial malignancy or other synchronous malignancy (such as GIST, esophageal cancer, or colorectal cancer); (6) no recurrent gastric cancer or remnant gastric cancer; and (7) no death in the perioperative period. Tumor resection and lymphadenectomy were performed by experienced surgeons, and the surgical procedures, which followed the Japanese Gastric Cancer Association (JGCA) guidelines, were similar in all patients who underwent radical resections.

The median age was 54±13 years (range 18–86). There were 130 males and 75 females (male:female = 1.733); the median male age was 54±12 years, and the median female age was 54±14 years.

### Statistical analysis

Statistical analysis was performed using the SPSS 13.0 statistical software. The measured data were denoted as the mean standard deviation (SD) and verified using t-tests. The numerical data were analyzed using Pearson’s Chi-square and Fisher’s exact probability tests. All 205 EGC cases were included in the Chi-square test and logistic regression analysis to investigate the relationship between clinicopathological parameters and LNM. Moreover, the survival data were analyzed using Kaplan-Meier and multiple Cox regression analysis. The survival curves were drawn, and the differences were verified using Log-rank tests. The differences were considered significant at P <0.05.

## Results

### Surgery and lymph node dissection

One hundred seventy-one patients underwent distal gastrectomy. Eighteen patients underwent total gastrectomy, and 16 underwent proximal gastrectomy. Lymph node dissections were D2 in 181 cases, and they were D1 in the remaining 24 cases. The overall survival rates in the D2 and D1 groups were not significantly different (P = 0.479). A total of 4,499 lymph nodes were resected, and 145 were found to be positive for cancer. Stratified analysis showed that the overall survival rate in patients with at most 15 resected lymph nodes was similar to that in patients with more than 15 resected lymph nodes (P = 0.360).

### Pathological findings

Post-operative pathological examination identified 97 (47.3%) cases of intra-mucosal cancer and 108 (52.7%) cases of sub-mucosal cancer. LNM occurred in 52 (25.37%) cases, with 35 (17.07%) cases staged as N1, 11 (5.37%) cases as N2 and 6 (2.93%) cases as N3. Additionally, we found 18 LNM cases occurred in intramucosal cancers (13 N1, 4 N2 and 1 N3), while 34 occurred in submucosal cancers (22 N1, 7 N2 and 5 N3). There were 8 (3.90%) cases of lymphatic vessel invasion and 2 (0.98%) cases of blood vessel invasion. Regarding the tumor position, 141 (68.78%) tumors were located in the gastric antrum, 39 (19.02%) in the gastric body and 25 (12.20%) in the gastric fundus or cardia. Seventy-eight (37.05%) cases had tumors larger than 3 cm, while 127 (61.95%) cases had tumors smaller than 3 cm in diameter. Of the latter, two cases were diagnosed as one-point cancer, and no micro-focus less than 0.5 cm in diameter was found. According to histological classification, there were 66 well-differentiated (12 cases) and mid-differentiated adenocarcinoma (54 cases), while the remaining 139 samples were diagnosed as poorly-differentiated adenocarcinomas (98 cases) or signet ring cell carcinoma (41 cases). According to the standards of the Japanese Society of Gastrointestinal Endoscopy (1962) and the Japanese Association of Gastric Cancer (1998), the general shapes could be classified into three types: 9 (4.39%) cases of the protruded type (Type I), 66 (32.20%) cases of the flat type and 130 (63.41%) cases of the excavated type (Type III). The flat type included 3 (4.54% of all Type II) cases of the elevated type (Type IIa), 30 (45.45%) cases of the superficial flat-type (Type IIb), and 33 (50%) cases of the superficial excavated type (Type IIc).

### Relationship between clinicopathological parameters and LNM

EGC is most frequently observed in patients aged between 41 and 60 years of age. The median age of the LNM-positive group was 52±13 years; no significant difference in age was observed in the LNM-negative group (P = 0.242, t-test). The incidence of LNM correlated significantly with gender (P = 0.046), tumor size (P = 0.017), depth of tumor infiltration (T1a or T1b, P = 0.034), tumor cell differentiation (P = 0.001) and lymphatic or blood vessel invasion (P = 0.003), but it was not correlated with age, tumor location, pre-operative status of CEA, CA724 and CA199, or general shape (Table 1). Logistic regression analysis demonstrated that tumor cell differentiation (P = 0.002) and the depth of tumor infiltration (P = 0.004) were the most important factors associated with EGC lymph node metastasis, followed by vessel invasion (P = 0.012), tumor size (P = 0.020) and gender (P = 0.022), listed in order of priority (Table 2).

**Table 1:** Correlation between LNM and clinicopathological factors of 205 EGC patients.

Clinicopathological parameters	*na*	LNM		χ^2^	*P Value*
		Positive	Negative		
**All**	205	52	153		
**Age (years)**					
<55	105	31	74	1.966	0.161
≥55	100	21	79		
**Gender**				3.966	0.046*
Male	130	27	103		
Female	75	25	50		
**Tumor size**				5.690	0.017*
<3 cm	127	25	102		
≥3 cm	78	27	51		
**Tumor location**				4.802	0.280
Fundus /cardia	25	2	23		
Body	39	11	28		
Antrum	141	39	100		
**Tumor infiltration**				4.509	0.034*
T1a	97	18	79		
T1b	108	34	74		
**Differentiation**				11.201	0.001*
Well/mid	66	7	59		
Poorly/signet ring cell	139	45	94		
**General shape**					
Protruded	9	2	7	2.361	0.307
Flat	66	21	45		
Excavated	130	29	101		
**CEA (ng/ml)**				2.552	0.110
≤5	183	50	133		
>5	22	2	20		
**CA199 (U/ml)**				2.478	0.115
≤35	194	47	147		
>35	11	5	6		
**CA724 (U/ml)**				1.575	0.210
≤5	183	44	39		
>5	22	8	14		
**Vessel invasion**				8.723	0.003*
Positive	10	7	3		
Negative	195	45	150		

^a^ Numbers of cases in each group.

* Statistically significant (*P*<0.05)

**Table 2:** Multinominal logistic regression analyses of LNM and interrelated clinicopathological factors.

Interrelated Clinicopathological parameters	*OR*	95%CI of OR		χ^2^	*P* Value
		Lower	Upper		
**Gender**				5.284	0.022*
Male	1.000				
Female	0.426	0.206	0.882		
**Tumor size**				5.401	0.020*
<3 cm	1.000				
≥3 cm	2.364	1.144	4.884		
**Tumor infiltration**				8.336	0.004*
T1a	1.000				
T1b	3.079	1.435	6.608		
**Differentiation**				9.185	0.002*
Well/mid	1.000				
Poorly/signet ring cell	4.125	1.650	10.314		
**Vessel invasion**				6.346	0.012*
Negative	1.000				
Positive	6.785	1.530	30.096		

OR, Odds ratio; CI, confidence interval;

* Statistically significant (*P* < 0.05).

### Relationship between clinicopathological parameters and EGC prognosis

**Fig 3:**
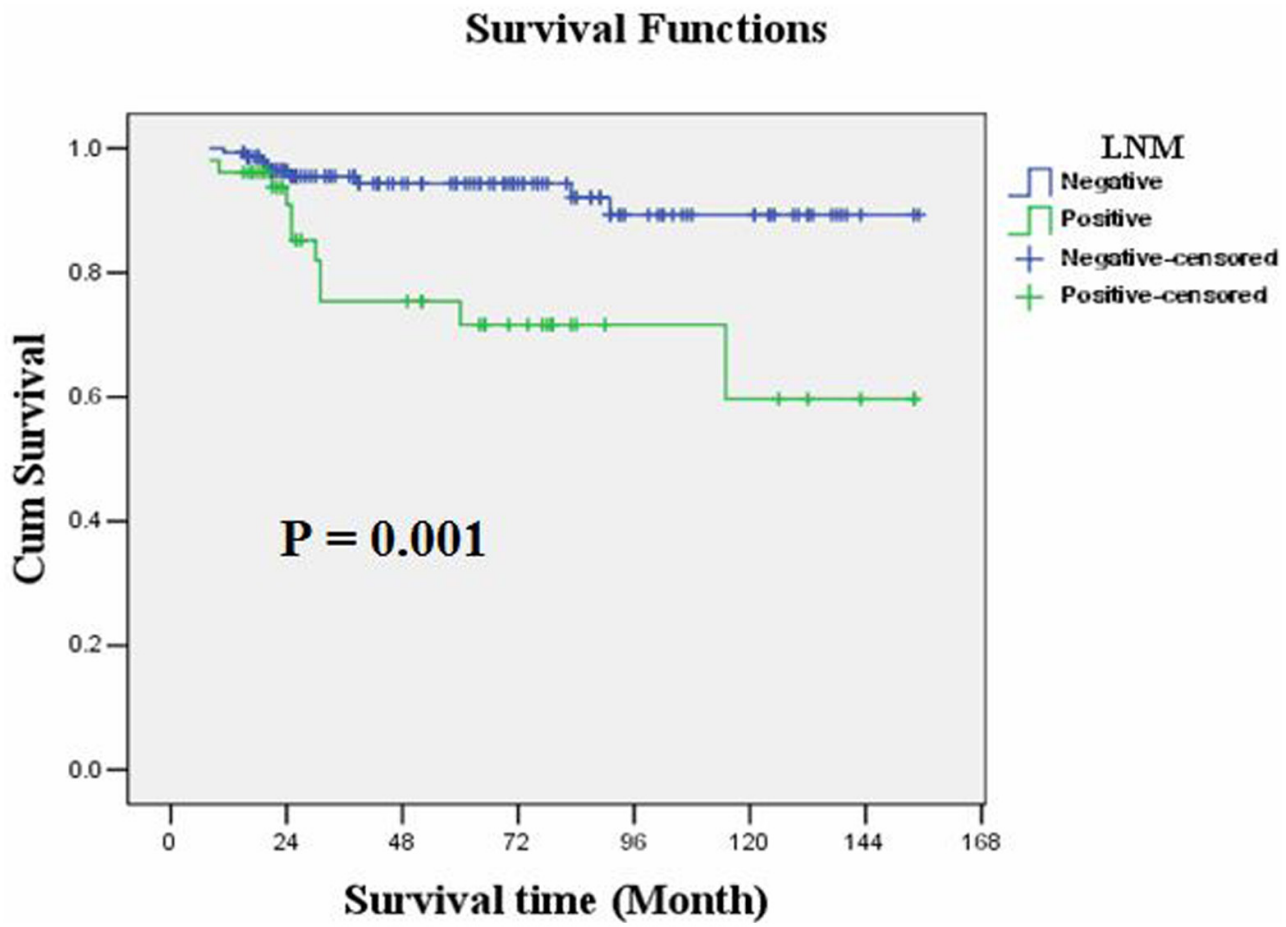
Kaplan-Meier survival analysis showed that overall survival of EGC was significantly correlated with LNM (P = 0.001).

**Fig 4:**
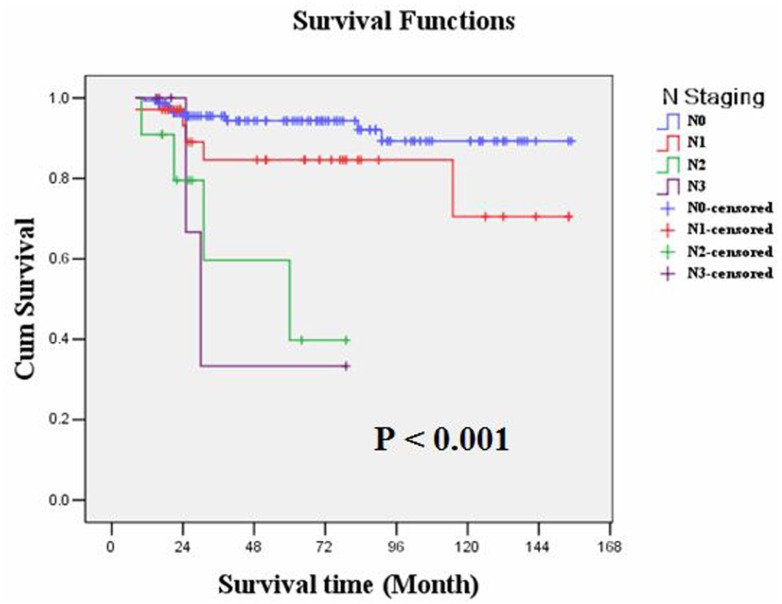
Kaplan-Meier survival analysis showed that overall survival of EGC was significantly correlated with N staging (P<0.001).

**Fig 5:**
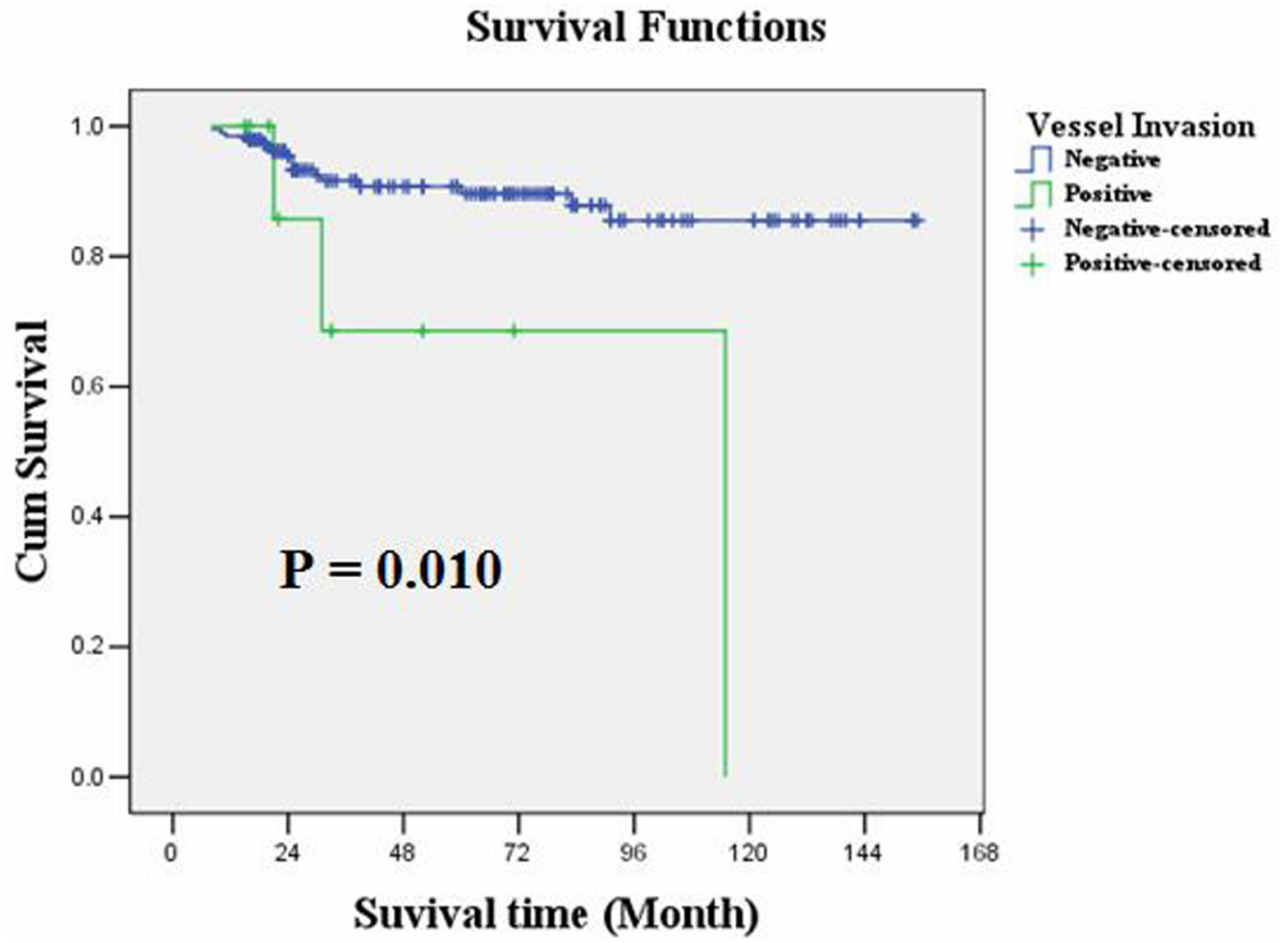
Kaplan-Meier survival analysis showed that overall survival of EGC was significantly correlated with invasions of lymphatic or blood vessels (P = 0.010).

**Fig 6:**
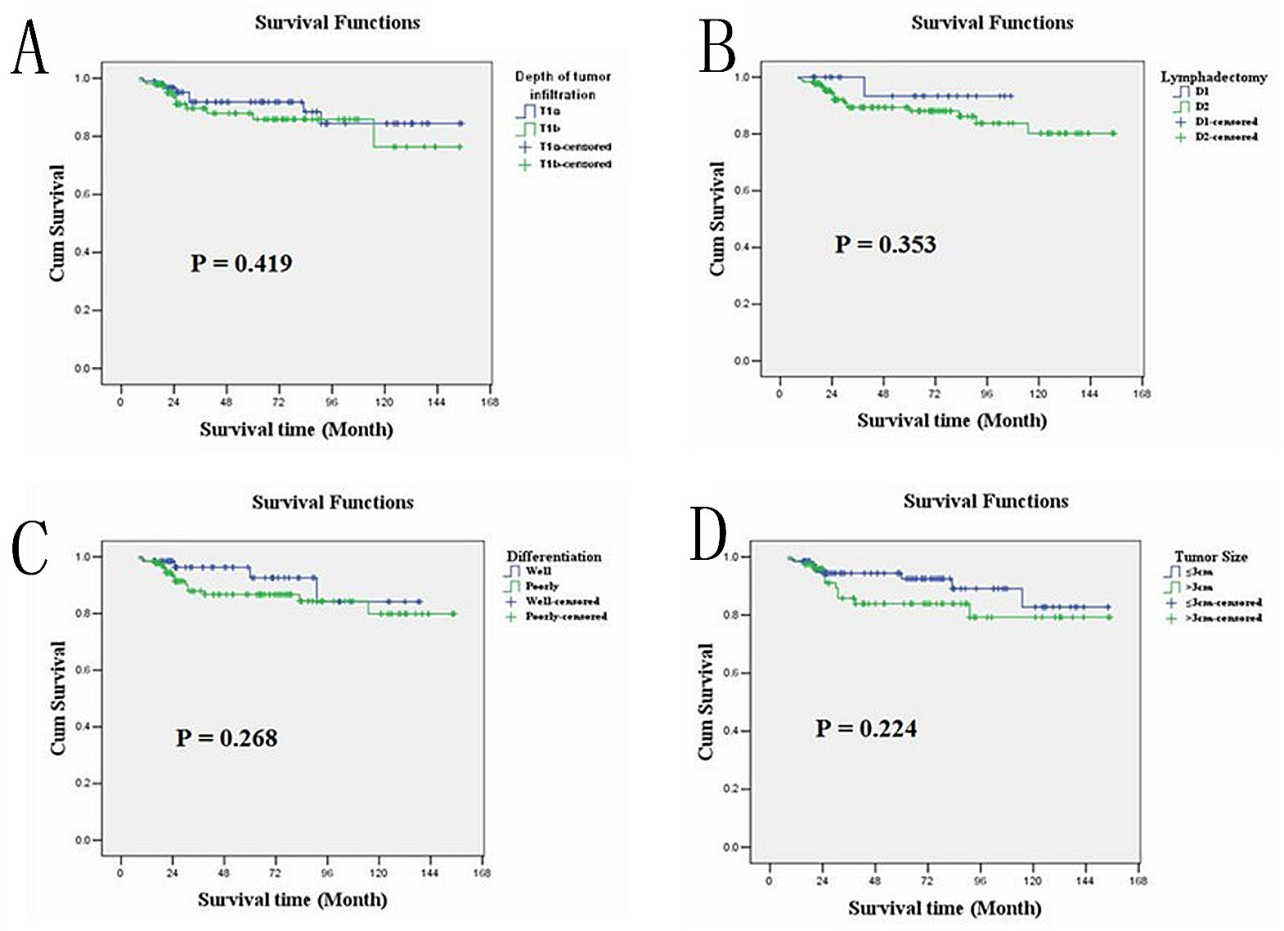
Kaplan-Meier survival analysis showed that overall survival of EGC was not correlated with (A) depth of tumor infiltration(P = 0.419), (B) D1 or D2 resection(P = 0.353), (C) cell differentiation(P = 0.268), (D)tumor size(P = 0.224).

The overall survival rate was 90.2%. Kaplan-Meier survival analysis showed that overall survival was significantly correlated with LNM (P = 0.001) or N staging (P<0.001) and invasions of lymphatic or blood vessels (P = 0.010), but it was not correlated with age, gender, tumor size, tumor location, depth of tumor infiltration, tumor cell differentiation or general shape. As with treatment, the overall survival of these patients showed no obvious relationship with the scope of gastric resection (P = 0.856), D1 or D2 operation (P = 0.353) or the number of lymphadenectomies (<15 or ≥15, P = 0.269). Moreover, multiple Cox regression analysis identified that only N staging (P = 0.001) could serve as an independent prognostic predictor in the EGC patients (Table 3). The overall survival rates of the LNM-negative and LNM-positive groups were 94.1% and 78.8%, respectively (P = 0.001, Log-rank test); the average overall survival time was 144.142 and 113.876 months, respectively. Kaplan-Meier plots are shown in Figs 3, 4, 5 and 6.

**Table 3:** Univariate and multivariate analyses of overall survival of EGC.

Variables	*n* ^a^	Kaplan-Meier survival analysis			Multiple Cox regression analysis		
		MS (Months)	(95% CI)	*P* Value	HR	(95% CI)	*P* Value
**All**	205	136.445	128.688–144.201				
**Age (years)**				0.150			
<55	105	142.290	133.867–150.713				
≥55	100	127.116	112.789–141.442				
**Gender**				0.327			
Male	130	133.911	123.335–144.487				
Female	75	139.311	128.010–150.611				
**Tumor size**				0.224			
<3 cm	127	139.699	130.499–148.898				
≥3 cm	78	131.292	118.440–144.414				
**Tumor location**				0.921			
Fundus /cardia	25	142.480	127.174–157.786				
Body	39	142.183	128.302–156.064				
Antrum	141	133.946	124.491–143.400				
**Tumor infiltration**				0.419			
T1a	97	139.251	128.833–149.670				
T1b	108	132.644	120.915–144.373				
**Differentiation**				0.268			
Well/mid	66	134.159	116.716–138.453				
Poorly/signet ring cell	139	127.584	116.716–138.453				
**General shape**				0.658			
1	9	115.514	85.432–145.597				
2	66	133.536	122.715–144.357				
3	130	142.026	131.211–152.841				
**Vessel invasion**				0.010*			0.177
Negative	195	138.970	131.637–146.304		1.000		
Positive	10	87.171	47.717–126.626		2.460	0.666–9.089	
**LNM**				0.001*			
Positive	52	113.876	93.984–133.767				
Negative	153	144.142	137.140–151.143				
**N Staging**				<0.001*			0.001*
0	153	144.142	137.140–151.143		1.000		
1	35	128.370	108.365–148.375		0.103	0.021–0.507	
2	11	52.813	33.519–72.106		0.218	0.040–1.176	
3	6	44.667	17.097–72.263		0.920	0.167–5.058	
**Resection scop of stomach**				0.856			
Distal	171	135.135	126.527–143.742				
Proximal	16	145.882	68.960–86.040				
Total	18	77.50	130.447–161.318				
**D1 or D2**				0.353			
D1	24	102.467	93.883–111.051				
D2	181	135.351	126.997–143.705				
**Number of lymphadenectomy**				0.269			
<15	45	129.907	112.110–147.705				
≥15	160	138.178	129.759–146.579				

MS, median survival; HR, hazard ratio; CI, confidence interval;

^a^ Numbers of cases in each group;

* Statistically significant (*P* < 0.05).

## Discussion

Gastric cancer (GC) is a common malignant tumor worldwide. With an estimated number of one million new cases every year, GC ranks as the fourth most common cancer [9]. EGC, defined as an intra- and sub-mucosa tumor with or without LNM, has a relatively higher overall survival rate (more than 90%) than advanced gastric cancer (ACG)[1]. Adachi et al reported in their review [4] that LNM occurred more frequently in sub-mucosa GC than in intra-mucosa GC (15% VS 3%). Moreover, they demonstrated the prognostic value of LNM in EGC patients. In recent decades, many post-operation survival analyses on EGC showed that intra-mucosal cancer without LNM could be cured by endoscopic mucosal resection (EMR), while other EGC patients required gastrectomy with D1 or D2 lymphadenectomy [7,10]. These data suggested that in clinical practice, accurate pre-operation assessments of EGC patients (mainly the depth of tumor infiltration and LNM) were critical in choosing optimal treatments. Therefore, the present study aimed to find an effective method to predict the existence of LNM in EGC patients to support new evidence to indicate the best treatment.

We retrospectively analyzed clinicopathological data from 205 EGC patients who underwent surgical resection at Sun Yat-sen University Cancer Center from January 2000 to December 2011. The main complaints of these patients included upper abdominal pain (75.12%), bloating (21.95), acid reflux (11.22%) and belching (7.32%). According to our data, most gastric cancer patients have a delayed diagnosis, as a result of lack of general gastroscopy survey. The tumor invaded deeper or progressed into AGC without obvious clinical manifestations. Therefore, sub-mucosa cancer accounts for a larger part of EGC in our center (108/205) than it does in Japan and Korea [11–12].

Our results showed that LNM occurred in 52 of the 205 EGC cases (25.37%). Of these 52, 18 were found in the 97 T1a cases (18.56%), and 34 were found in the 108 T1b cases (31.48%). These occurrences are significantly higher than the 10% lymph node positivity ratio, which was reported by Saka et al [13] based on an analysis of 2,368 EGC cases recorded in the Japan National Cancer Center. We can hypothesize that EGC in China has its own features, such as deeper tumor invasion and more LNM. Then, we further analyzed the correlation between LNM and the clinicopathological factors of these patients and found that gender (P = 0.046), tumor size (P = 0.017), depth of tumor infiltration (T1a or T1b, P = 0.034), tumor cell differentiation (P = 0.001) and lymphatic or blood vessel invasion (P = 0.003) correlated with LNM. Moreover, a logistic regression analysis demonstrated that tumor cell differentiation (P = 0.002) and depth of tumor infiltration (P = 0.004) were the most important factors associated with EGC lymph node metastasis, followed by vessel invasion (P = 0.012), tumor size (P = 0.020) and gender (P = 0.022). Female EGC patients suffered a higher LNM ratio than the male EGC patients did (33.33% vs. 20.77%); LNM was more likely to correlate with large tumors. Additionally, 21 of the 41 cases with tumors larger than 4 cm were positive for LNM (47.73%), while only 1 of the 13 cases with tumors smaller than 1 cm was positive for LNM. As with tumor differentiation, 13 of the 34 (38.24%) signet ring cell cancers were positive for LNM. Similar to our study, most previous studies [14–16] concluded that the depth of tumor infiltration, or T1a/T1b, is the most important factor correlated with LNM. Our data were consistent with that conclusion, and we further discovered that there were 13 N1 cases, 4 N2 cases and only 1 N3 case of T1a, while there were 22 N1, 7 N2 and 5 N3 cases of T1b. These data indicated that the depth of tumor invasion was correlated not only with the incidence rate of LNM but also with the degree or number of metastatic lymph nodes. However, LNM incidence showed no significant differences in terms of age, tumor location, general shape or the pre-operative statuses of CEA, CA199 and CA724. In all, poorly differentiated cells, the tumor invading into the sub-mucosa layer, tumor size larger than 3 cm, vessel invasion and being female were the risk factors of LNM, listed in order of priority. Four of these risk factors (excluding vessel invasion) are detectable and can be used to predict LNM. According to our results, EGC patients with only one risk factor had a 31.5–34.6% incidence rate of LNM, patients with two risk factors had a 38.9–47.1% rate, patients with three risk factors had a 53.8–66.7% rate and patients with all four risk factors had a more than 80% incidence rate of LNM. Interestingly, in this study, 15 patients had no risk factor (including vessel invasion), and 6 patients had all four risk factors. None of the 15 risk-free patients had LNM, while all 6 highest risk patients had LNM. These data may provide a reference for pre-operative assessment and an optimal choice for EGC treatment.

EGC has a better prognosis than AGC. A recently study reported that the 5- and 10-year survival rates for EGC are more than 90% and 80%, respectively [17]. The current study identified the median survival (MS) time for EGC patients as 136.445 months (95CI:128.688–144.201). Using Kaplan-Meier survival analysis, we demonstrated that vessel invasion and N staging (lymph node metastasis) are important prognostic predictors for EGC. Multiple Cox regression analysis showed that only N staging could independently predict the prognosis of EGC patients. Inconsistent with previous studies [18], we found that N staging was the only independent prognostic factor and that tumor size, tumor cell differentiation and depth of tumor infiltration could not independently predict EGC patient prognosis. The four above-mentioned clinicopathological factors correlated with LNM and therefore could not directly predict EGC prognosis. There was no significant difference in the overall survival of T1a and T1b patients (139.251 vs. 132.644). Moreover, all patients in this study underwent D1 or D2 lymph node dissection, and we found no significant differences in their survival. There was also no significant difference in the survival rates for patients who underwent lymphadenectomy (<15 or ≥15).

How to make the best choice among the possible treatments for EGC, including EMR/ESD [19], laparoscopic gastrectom [20]and traditionalD1 or D2 radical resection [21], remains debatable. Pre-operative LNM status will certainly be useful information when making a clinical decision. However, clinical risk factors can only be used to roughly evaluate the incidence of LNM. The development of accurate methods requires new biomarkers. In recent decades, several studies related to the discovery of new biomarker has been performed. Tamura Y showed that MUC4 and MUC1 were correlated with LNM and had the potential to be new markers for the prediction of LNM in EGC [22].

Better survival depends on the early diagnosis and accurate pre-operative assessment of EGC. The results of the current study suggest that female patients with sub-mucosa invasion, tumors larger than 3 cm in diameters, poorly differentiated cells and lymphatic or blood vessel invasion are at high risk of LNM and have poor prognosis. Treatment, including surgical resection and other adjuvant therapies, should be cautiously planned, and these clinicopathological factors should be considered.
